# Reversing Autoimmunity Combination of Rituximab and Intravenous Immunoglobulin

**DOI:** 10.3389/fimmu.2018.01189

**Published:** 2018-07-18

**Authors:** A. Razzaque Ahmed, Srinivas Kaveri

**Affiliations:** ^1^Department of Dermatology, Tufts University School of Medicine, Boston, MA, United States; ^2^Center for Blistering Diseases, Boston, MA, United States; ^3^INSERM U1138 Centre de Recherche des Cordeliers, Paris, France

**Keywords:** reversal of autoimmunity, autoimmune diseases, autoantibodies, B cell development, B cell depletion therapy, intravenous immunoglobulin, rituximab, autoimmune blistering diseases

## Abstract

In this concept paper, the authors present a unique and novel protocol to treat autoimmune diseases that may have the potential to reverse autoimmunity. It uses a combination of B cell depletion therapy (BDT), specifically rituximab (RTX) and intravenous immunoglobulin (IVIg), based on a specifically designed protocol (Ahmed Protocol). Twelve infusions of RTX are given in 6–14 months. Once the CD20^+^ B cells are depleted from the peripheral blood, IVIg is given monthly until B cells repopulation occurs. Six additional cycles are given to end the protocol. During the stages of B cell depletion, repopulation and after clinical recovery, IVIg is continued. Along with clinical recovery, significant reduction and eventual disappearance of pathogenic autoantibody occurs. Administration of IVIg in the post-clinical period is a crucial part of this protocol. This combination reduces and may eventually significantly eliminates inflammation in the microenvironment and facilitates restoring immune balance. Consequently, the process of autoimmunity and the phenomenon that lead to autoimmune disease are arrested, and a sustained and prolonged disease and drug-free remission is achieved. Data from seven published studies, in which this combination protocol was used, are presented. It is known that BDT does not affect check points. IVIg has functions that mimic checkpoints. Hence, when inflammation is reduced and the microenvironment is favorable, IVIg may restore tolerance. The authors provide relevant information, molecular mechanism of action of BDT, IVIg, autoimmunity, and autoimmune diseases. The focus of the manuscript is providing an explanation, using the current literature, to demonstrate possible pathways, used by the combination of BDT and IVIg in providing sustained, long-term, drug-free remissions of autoimmune diseases, and thus reversing autoimmunity, albeit for the duration of the observation.

## Introduction

The treatment of autoimmune disease is a challenge and priority as they affect 50 million individuals in the United States, and currently they collectively are the third most common disease category (1, 2). This importance is further emphasized by the fact, that many people may be affected for a significant portion of their lives (1, 2). Historically, their prognosis dramatically improved with the advent and use of systematic corticosteroids (CS). Unfortunately, the much-needed long-term use of high dose systemic CS resulted in serious, and catastrophic, side effects, which caused morbidity and mortality (3). Consequently, immunosuppressive agents (ISA) in combination with CS became the major treatment modality (3, 4). The long-term use of this combination resulted in profound immune suppression, resulting in fatal opportunistic infections. B cells are considered the key participants in autoimmune diseases, since they produce pathogenic autoantibodies (5). Recently, B cell depletion therapy (BDT), specifically rituximab (RTX), has gained enormous popularity in the treatment of autoimmune diseases (6, 7).

B cell depletion therapy produces rapid clinical remissions in 70–80% of patients (8). However, majority if not most patients, develop relapses. Benefits from a single infusion usually last 9–18 months (9, 10). The reported rate of relapse depends on the duration of follow-up (11–15). As the effect of the BDT wears off clinical disease recurs, suggesting that BDT may not produce long-term sustained clinical remissions, without its continued use.

## Unmet Needs

The preferable drug(s) to treat autoimmune disease would be whose use requires a defined protocol, grounded in scientific fact, with predictors of progress, discernible end point, produces long-term sustained clinical remission, without need for continued or additional therapy. It must be safe, have minimal immediate and long-term adverse events, readily available, easy to administer, and produce a good quality of life. Currently, no such drugs or agent(s) are available. To fulfill this unmet need, based on scientific literature, presented in this manuscript, a protocol was developed, and is the focus on this paper.

## The Concept

Autoimmune diseases that are associated with the autoantibodies are presumed to be B cell driven (5). Therefore, BDT was used. Since use of RA protocol, in which 1 g of RTX is given 15 days apart, was associated with high relapse rates, multiple infusions were given (12). The rationale being that multiple infusions may delete CD20^+^ B cells (10). Repeated infusions would capture those cells residing at immune privileged sites, subsequently leave, enter peripheral circulation, and are lysed (16). Removal of autoreactive B cells would decrease inflammations in the microenvironment and eventually reduce pathogenic autoantibody production (9).

Intravenous immunoglobulin has multiple effects on the immune and inflammatory pathways (17–19). The properties used in this concept are those that act synergistically with BDT. IVIg would enhance and complement the anti-inflammatory effects of BDT (20–22). Once inflammation is significantly reduced or eliminated, IVIg might restore immune balance to normal homeostasis (23, 24). During BDT induced B cells depletion, IVIg provides immunoprophylaxis. Simultaneously, the multiple IVIg infusions produce sustained long-term clinical remissions, decrease and possibly eliminate pathogenic autoantibody (25).

Commercial preparations of IVIg in addition to IgG, contain small amounts of several molecules, playing a vital role in its biologic and pharmacological functions (24). Many have not been identified, and amongst others include cytokines, cytokine receptors, molecular markers, antibodies against T cells determinants, such as CD4 CD8, HLA antigens, super antigens, and anti-idiotypic antibodies (24). Their amounts are not constant and vary among batches, from the same manufacturer. Their relative amounts vary among brand names, since the patents used, have different biochemical methodologies and industrial processes (26). One of clinical implications of these variabilities is that, patients need multiple infusions to demonstrate clinical benefit, and subsequently additional infusions to retain the benefit obtained. Critical level of biochemical molecules or receptors may only be obtained from multiple infusions. Single or limited infusion may not provide the pharmacodynamics necessary for producing desired clinical response. Another explanation for multiple infusions is that antibodies in commercial preparations compete with pathological autoantibodies for neonatal Fc receptor (FcRn) binding (27). One recent clinical study highlights the long-term benefits of multiple infusions. In patients with chronic inflammatory demyelinating polyneuropathy, limited infusions of IVIg provide significant clinical relief because of its anti-inflammatory effect. Durations of remission are limited, relapse occurs, requiring repeat infusion (28). Recent study demonstrated that multiple infusions during the post-inflammatory recovery period provided prolonged remission lasting up to 52 weeks, in 70% of patients (28). This study demonstrated that it was multiple infusions, at defined intervals, after clinical recovery that produced a sustained prolonged clinical remission (28).

## The Protocol

**Figure 1:**
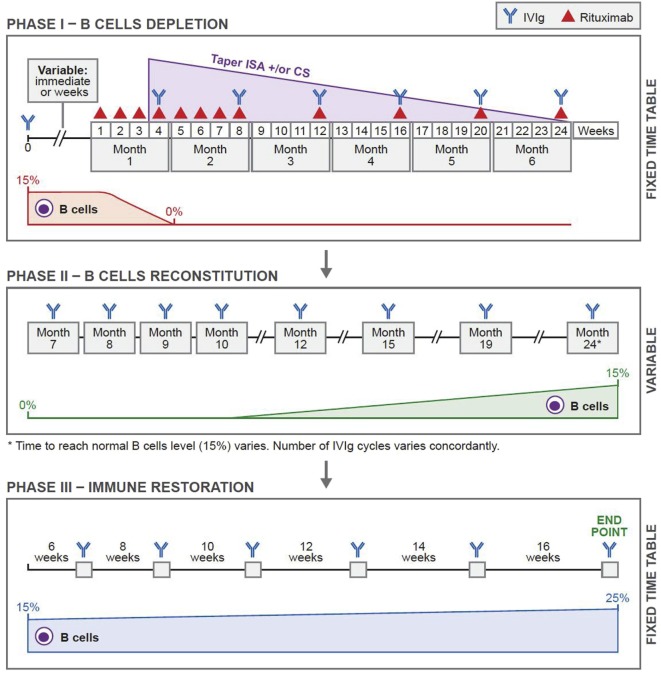
Novel protocol using a combination of rituximab (RTX) and intravenous immunoglobulin (IVIg). In phase 1, before initiating RTX, patients were given one cycle of IVIg (2 g/kg/cycle) for immunoprophylaxis. Thereafter, they were given weekly infusions of RTX (375 mg/m^2^) for eight consecutive weeks. Additional RTX infusions were given once a month for four consecutive months. Thus, the patients received 12 infusions of RTX during a 6-month period. IVIg was given at monthly intervals during phase 1 for continuing immunoprophylaxis. In phase 2, patients received multiple cycles of IVIg at monthly intervals. The number of cycles depended on the repopulation of B cells. The CD19^+^ B-cell count was 0 at the beginning. Phase 2 ended when the CD19^+^ B-cell count was 15%. Phase 3 was based on a published protocol and was consistent in all patients. Each patient received a total of six cycles of IVIg given at 6-, 8-, 10-, 12-, and 14-week intervals. The last cycle was at 16-week interval. Thus phase 3 lasted 16.5 months. This was the end of the protocol. Abbreviations: CS, corticosteroids; ISA, immunosuppressive agents. Reprinted from Ref. (29), with permission from Elsevier.

The protocol that has produced sustained clinical remissions without need for subsequent systemic therapy is presented in Figure 1 (Ahmed Protocol) (29). It has three phases. In phase 1, patient received a cycle of IVIg before receiving RTX, then eight infusions of RTX in eight consecutive weeks, at a dose of 375 mg/m^2^ per infusion. Thereafter, four additional infusions of RTX either at monthly or preferably at 3 months interval are administered. Patients are disease free during this period. These repeated infusions will enhance the penetration of RTX into bone marrow (BM), lymph nodes (LNs), and spleen, eliminates those CD20^+^ B cells that freshly migrate into the peripheral blood (PB) from these sites. The CD20^+^ B cell count is zero usually by the second or third infusion. Since the half-life of IVIg is usually 3 weeks, it is administered monthly (30). During phase 1, previous initiated treatments with CS and ISA have no demonstrable pharmacologic benefit or need, are tapered and discontinued.

Phase 2, is the period of continued B cell depletion. IVIg infusions are continued until B cells repopulate.

In phase 3, IVIg is used according to an established protocol (31), developed by a Consensus Development Committee on the use of IVIg therapy for the treatment of autoimmune mucocutaneous blistering diseases, in which it initially produced 36 months sustained remissions, without any additional systemic therapy (31, 32). Twenty years later, these patients are still in remission (unpublished data). In these patients, pathogenic autoantibody levels declined and were non-detectable during follow-up periods (33–38). These results were demonstrated in patients with pemphigus vulgaris (PV), pemphigus foliaceus, bullous pemphigoid (BP), mucous membrane pemphigoid (MMP) aka cicatrical pemphigoid, oral pemphigoid, ocular cicatrical pemphigoid (OCP), epidermolysis bullosa acquisita (EBA), and pemphigoid gestationis (33–38). Collectively, the data strongly suggest that, these last seven infusions during phase 3, most likely affected multiple cells, molecules, mediators, and receptors, causing a restoration of immune balance, return to hemostasis and possibly re-institution of tolerance.

The beneficial effects of IVIg, its anti-inflammatory actions and influence on the microenvironment, are best understood from situations where clinical failure or lack of response was observed. In such situations, the inflammatory influences are excessive. Wherein the microenvironment does not allow IVIg to exert immune-regulatory effects. Beneficial effects of IVIg can be influenced by mediators of inflammation, such granulocyte-colony stimulating factor (G-CSF), IL-1β, and IL6 (39). Threefold higher levels of G-CSF were observed in patients nonresponsive to IVIg compared to responders. G-CSF uses signaling pathways of STAT3 for induction of granulopoiesis and differentiation of granulocytes (39). Resultant high levels of neutrophils in the microenvironment, possibly inhibit many functions of IVIg. Interestingly, BDT can significantly inhibit STAT3 activity, resulting in reduction of granulopoiesis, neutrophil production, and decrease in inflammation in the microenvironment of the tissues (40). This supports the concept of synergistic effects of RTX and IVIg.

## Proof of Concept

The authors combined RTX with IVIg in a specific protocol (Ahmed Protocol) (Figure 1). All patients that are reported by our group completed the protocol. Limited aspects, relevant features including the results of treatment of severe recalcitrant patients with pemphigus vulgaris, bullous pemphigoid, mucous membrane pemphigus, ocular cicatrical pemphigoid, and epidermolysis bullosa acquisita (EBA) are presented in Table 1 (29, 41–45).

**Table 1:** Relevant data from autoimmune bullous diseases treated with rituximab (RTX) and intravenous immunoglobulin (IVIg).

	Pemphigus vulgaris		Bullous pemphigoid	Mucous membrane pemphigoid	Epidermolysis bullosa aquisita
No of patients	11	10	12	6	5
Mean age at onset	38 (15–68)	47.8 (35–64)	68.25 (60–76)	74 (69–79)	50.6 (31–61)
Mean duration of disease before combined therapy	68.8 months (32–219 months)	None	36.5 months (2.7–72 months)	7 years (4.5–9.6 years)	8.8 years (2–20 years)
Duration of combined therapy	28 months (22–40 months)	32 months (17–52 months)	23.8 months (20–30 months)	24.6 months (18–29 months)	25 months (18–23 months)
Time to B cell depletion	4 weeks (3–6)	1.5 weeks (0.5–2.5)	2.3 weeks (0.5–3.9)	1.8 weeks (0.5–3.1 weeks)	2.0 weeks (0.6–3.4 weeks)
Time to B cell repopulation	12 months (18–26 months)	22.6 months (14–28 months)	70 months (17–31 months)	22 months (16–36 months)	18 months (12–28 months)
Time to serological remission	18 months (14–22 months)	11 months (9–14 months)	14 months (10–18 months)	14 months (12–18 months)	N/A
Clinical outcome	CCR	CCR	CCR	CCR	CR
SAE infection	None	None	None	None	None
SAE cardiac	None	None	None	None	None
SAE deaths	None	None	None	None	None
Mean total duration of follow-up	132.5 months (15–37 months)	131.7 months (111–136 months)	73 months (48–144 months)	9.8 years (99–115 months)	26 months (10–29 months)
Relapse reported, since discontinuation of combined treatment	None	None	None	None	None

*CCR = complete clinical remission = no disease, no drug*.

*CR, clinical remission on therapy; NA, not available*.

Into October 2006, we reported successful clinical outcomes in 11 patients with severe widespread PV involving the skin and multiple mucosae (41). These patients were previously treated with long-term high dose prednisone, 4–8 ISA and IVIg. The duration of all systemic therapy prior to combination therapy was mean of 68.8 months (range 31–219). The mean duration of follow-up after discontinuation of combination therapy was a mean of 31.7 months (range 22–37). None of the patients had any serious infection, were hospitalized for disease- or treatment-related issues, and remained in complete clinical remission, off therapy, with no detectable autoantibody and normal B cells.

In 2015, follow-up study reported that the patients continued to remain in complete clinical remission, off systemic therapy, with serological and immunopathological remission, and normal levels of B cells (42). The serum IgM levels reduced after a RTX therapy, became normal. They were carefully monitored, did not develop malignancies or other auto-immune disease. Hence, for 12–15 years post therapy a prolonged sustained clinical, serological, and immuno-pathological remission resulted from the combination therapy (42).

Ten patients with severe and widespread PV with contraindications for CS and ISA, treated with the combination of IVIg and RTX, using the same protocol, were reported in 2016 (43). Combination therapy was first-line treatment. Clinical response and complete disease resolution was rapid. During the follow-up of 131.7 months (10.97 years), the patients were in complete clinical remission, off therapy, with accompanying serological and immune-pathological remission, no relapses, infections, hospitalizations, or serious adverse events. An approximately, 10-year later the remissions remain.

Twelve patients with recalcitrant bullous pemphigoid (BP) who had failed CS and ISA, had multiple relapses on them, some had already received RTX by RA protocol, were treated by the combination of RTX and IVIg and followed for a mean of 73.8 months (6 years) post therapy were reported in 2015 (29). The patients remained in complete clinical remission off therapy, in serological and immune-pathological remission with normal levels of B cells in PB. They experienced no serious adverse events, infections, or hospitalizations and enjoyed a high quality of life. These remissions remain in a current follow-up of 9 years.

The combination of RTX and IVIg used to treat patients with recalcitrant ocular cicatrical pemphigoid (CP) and MMP has been reported in a controlled trial (44). These patients had MMP with multiple mucosal involvements, but the urgent issue was impending total blindness. 12 patients were divided into two groups. The control group had six patients, blind in one eye, with rapidly progressive OCP in the second eye, were treated with conventional therapy with ISA. All six patients became blind in both eyes. In the study group, four patients blind in one eye and two had persistent bilateral severe non-responsive conjunctival inflammation, were treated with RTX and IVIg. There best-corrected visual acuity and Foster OCP staging of the study group recovered and stabilized. Blindness was prevented. They had no serious adverse events to RTX or IVIg, no infections, no hospitalizations and a good quality of life. Initially followed for 1-year post therapy (44) and now for additional 8 years, these six patients with recalcitrant OCP, had complete clinical and serological remission, with normal levels of B cells, and a good quality of life and most importantly preventions of blindness.

Investigators from Turkey reported five patients with cutaneous and mucosal epidermolysis bullosa acquisita (EBA), resistant to conventional therapy, who were treated with combination of RTX and IVIg in 2017 (45). The published protocol for the combination of RTX and IVIg (29) was not followed. Modified protocol was used. Patients did have a positive clinical response, but still were under treatment 22 months post therapy.

A British group in 2016 reported three patients with MMP and one with linear IgA bullous disease, non-responsive to conventional therapy, all four blind in one eye, treated with two cycles of RTX and 2–9 monthly infusions of IVIg (46). Ahmed protocol (41) was not used. Visual acuity stabilized in all patients and progression of scarring ceased. Three of the four patients continued to require conventional immunosuppressive therapy after discontinuing IVIg and RTX. Follow-up post combination therapy was limited. One patient developed pneumonia followed by life-threatening septicemia and corneal infections, 3 and 6 weeks, respectively, after RTX infusions.

This rather limited sample of studies on autoimmune blistering disease, adds an extremely important dimension to the “Concept,” *albeit* a little early in the overall scheme. In the two studies (45, 46) in which the Ahmed protocol was not followed, the outcomes were not as favorable compared to outcomes where the protocol was followed (29, 41–44). These limited observations validate its potential to produce long-term sustained clinical and serological remission.

It needs to be highlighted that autoimmune mucocutaneous blistering diseases are used as for proof of concept, only because published data, though limited, was available, and complete to validate the principle. The data lack experiments that would have given a molecular and cellular basis to the concept. The purpose of the authors is to encourage other investigators to emulate the concept and use the Ahmed protocol. It is interesting to note that BDT has shown to be effective in autoimmune diseases generally considered to be T cell mediated, such as type 1 diabetes, multiple sclerosis, and thrombocytopenic purpura (TTP) (47–50).

Certain features in the clinical profile and course give evidence for recalcitrant disease. Diseases were present for several years, failed high doses of CS and multiple ISA, IVIg and in some one cycle of RTX, had turbulent clinical course with multiple relapses and remissions, and numerous significant or catastrophic side effects, resulting in frequent hospitalization, poor quality of life, and frequent loss of employment. Combination therapy was used as a treatment of last resort.

In addition to sustained long-term clinical remission, serological and tissue immunopathology, this combination has additional benefits. Patients with MMP, oral pemphigoid (OP), OCP, and EBA ceased to have disease progression.

The authors recognize that data presented have definitive limitations. Number of patients and diseases are limited. Studies are retrospective and lack controls. Control group of similar recalcitrant patients are difficult to obtain, specially is rare and orphan diseases. Controlled studies on such sick patients could be unethical.

## Discussion

The authors provide the molecular and cellular basis from relevant studies in the literature, to provide the basis for reported observations. The mechanism of action of IVIg, and the effects of BDT on the immune system are presented. To put the “concept” into proper perspective, certain features of autoimmunity and autoimmune disease are discussed, only to demonstrate how and where these biologic agents have the potential to influence them. The attempts of the authors are to demonstrate the mechanism by which the combination of IVIg and BDT influences the clinical course and positive outcome. Ultimately, there could be reversal of autoimmunity, *albeit* only for the duration of the reported follow-up period.

It needs to be emphasized that this discussion is not focused or targeted to a specific autoimmune disease. This data derived from multiple sources, human and animal, *in vitro* and *in vivo* studies, is solely to present cellular and molecular evidence for the “concept.”

The illustrations in this manuscript taken from publications, with the permission of the publishers, utilize known facts to provide a foundation for the “concept.” Legends accompanying the illustrations have been included because they contain messages and valuable information to understand immunology germane to the “concept.”

## Tolerance and Autoimmunity

A detailed and comprehensive discussion on tolerance and autoimmunity is beyond the scope of this manuscript. Limited and relevant literature on both, specifically those directly impacting the proposed “concept,” and possible mechanism involved in producing the positive clinical outcomes observed.

Two features are pivotal. First, the importance of the microenvironment and processes within it that influence disease manifestation, and response to therapy. Second, inflammation is central to the pathogenesis and persistence of autoimmune diseases. The best proof of this comes from the fact, that if not all, most autoimmune diseases respond to CS (3). Frequently, as doses are reduced or discontinued, relapses occur. When the dosages are increased, clinical remissions are restored.

Tolerance is a state of natural equilibrium, wherein the body does not permit development of detrimental phenomena, and eliminates whichever occur (51, 52). Alteration or loss of tolerance results in autoimmunity (53). The progression from autoimmunity to autoimmune disease is complex, and multi-factorial, dependent on autoreactive B cells, T cells, plasma cells, and many other cells and molecules involved in inflammatory pathways and the immune system. These include several cytokines, leukotrienes, lymphokines, growth factors, and signaling pathways (51–56).

### B Cell Development—Role of Checkpoints

**Figure 2:**
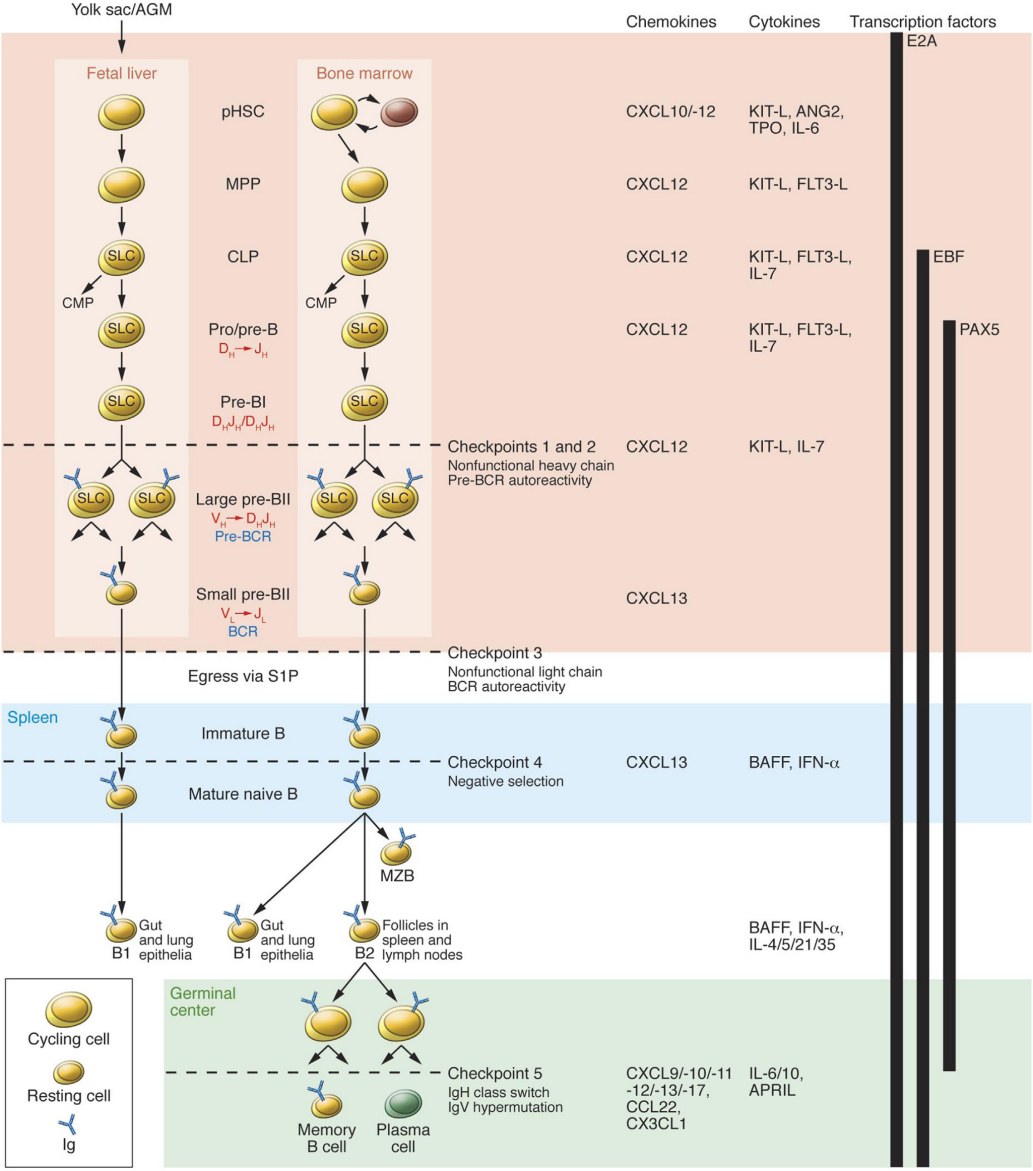
B cell development in fetal liver and bone marrow (BM). pHSC progenitors originate extraembyronically from yolk sac and, at later stages of development, intraembyronically from the aorta-gonad-mesonephros region. pHSCs develop in the fetal liver prenatally and in BM postnatally; both of these environments (pink region) provide crucial transcription factors, chemokines, cytokines, and cell contacts that regulate differentiation. Additionally, pHSCs localize to specialized niches that allow for their long-term survival in BM but not in fetal liver. Expression of the transcription factor E2A and the V(D)J rearrangement machinery RAG1/2 restricts CLPs from developing into B lineage cells. B cell receptors (BCRs) are generated by stepwise rearrangements of Ig segments. Pre-B cells that have undergone productive V_H_D_H_J_H_ rearrangement express an Igμ chain that can pair with SLC to form a pre-BCR, which stimulates large pre-B cell proliferation. lastly, V_L_-to-J_L_ rearrangement occurs in small pre-BII cells, which become surface BCR-expressing immature B cells. They then enter the spleen (blue region), where fetal liver-derived BI cells predominately populate the gut and lung epithelia as mature B cells. BM-derived B cells mature in spleen to BI cells, which populate the lung and epithelia; MZB cells, which populate the marginal zone; and BII cells, which are organized in B cell-rich follicles where T cell-dependent antigenic stimulation promotes development of germinal centers (green region). Antigen-specific follicular helper T cells induce B cell Ig class-switching and IgV-region hypermutation and help to develop memory B cells and plasma cells. The developing B cell repertoires are monitored for structural fitness and autoreactivity at five checkpoints. Abbreviation: CMP, common myeloid progenitor. Reprinted from Ref. (56) with permission from American Society for Clinical Investigation.

Checkpoints that play a significant role in the process of B cell tolerance have been characterized (56) (Figure 2). Checkpoints 1 and 2, located in the BM influence pre B cells and B cell receptor (BCR). At checkpoint 3, between BM and PB, reactivity of the BCR develops and can respond to self-antigens. At checkpoint 4, in the spleen, positive or negative selection of B cells occurs. At checkpoint 5, mature B cells undergo somatic hypermutation. Deletion of genes can downgrade serum light chains, leading to continued pre BCR expression, resulting in pre B cell hyperplasia. These mutations can influence emergent self IgH-repertoire, maintaining the presence of autoreactive pre B cells. At checkpoint 5 present in the germinal centers of the LNs and spleen. B cells encounter auto-antigens, in the context of the MHC class II gene products, and can possibly produce autoreactive memory B cells (57). Checkpoints 1, 2, and 3 are referred to as central tolerance and checkpoints 4 and 5 to as peripheral tolerance (56).

**Figure 3:**
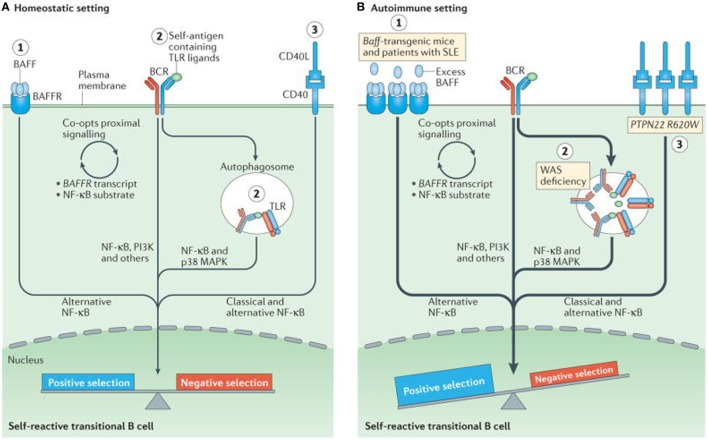
Altered B cell receptor (BCR) and co-receptor signaling promotes increased autoreactivity within the naive B cell repertoire. **(A)** Under homeostatic conditions, self-reactive B cells are subjected to both positive and negative selection mechanisms as they transit into the naive B cell pool and establish the naive repertoire. Whereas, tonic BCR signaling and BCR engagement with self-antigen primarily regulate these events, synergy between the BCR and co-receptors fine-tunes the tolerance program within a given B cell. Among these co-receptors, B cell activating factor receptor (BAFFR) signaling (1) synergizes with BCR signaling during late bone marrow and transitional development through a series of complex events, including proximal biochemical crosstalk and the downstream transcriptional regulation of both receptor and substrate expression. Dual BCR and toll-like receptor (TLR) signaling (2) is mediated by internalization and delivery of self-antigens that contain TLR ligands to autophagosomes, which contain endosome-resident TLRs. CD40 signaling (3), which is triggered by interaction with CD40 ligand (CD40L) on T cells and possibly other cell types, also integrates with the BCR signaling pathway. Although BCR signaling can modulate CD40 expression, other biochemical or transcriptional events that affect this crosstalk are less well understood. **(B)** In genetic (or environmental) settings that promote an increased risk of developing autoimmunity, the homeostatic signaling thresholds are modulated, and self-reactive B cells exhibit greater positive selection, and/or reduced negative selection, leading to a naive repertoire that is skewed toward autoreactivity. For example, excess amounts of B cell-activating factor (BAFF 1) in the *Baff*-transgenic mouse model rescue low-affinity self-reactive B cells from negative selection. A similar mechanism has been proposed to exist in individuals with systemic lupus erythematosus (SLE). Similarly, in mouse and human settings of Wiskott–Aldrich syndrome (WAS) deficiency (2), hyper-responsive dual BCR and TLR signaling promotes the positive selection of transitional B cells with BCRs that use a limited subset of genes that encode self-reactive heavy-chain variable (VH) domains. Healthy individuals with the autoimmunity-associated variant *PTPN22*^R620W^ (3) exhibit altered BCR and CD40 signaling, and have an enrichment of self-reactive BCR specificities within the naive B cell compartment. Although it has not yet been definitively demonstrated, it is likely that enhanced positive selection, rather than relaxed negative selection, predominantly mediates this change. The thickness of the arrows indicates the strength of pathway activation. Abbreviations: MAPK, mitogen-activated protein kinase; NF-κB, nuclear factor-κB; PI3K, phosphatidylinositol 3-kinase. Reprinted from Ref. (60) with permission from Springer Nature.

At checkpoints 4 and 5 there is a potential influence from toll-like receptor (TLR) (58, 59). Negative selection of autoreactive B cells prevents autoreactivity by process of deletion, receptor editing, and anergy. In contrast, positive selection occurs due to clonal expansion of transitional B cells, in the periphery and is affected by interaction with BCR signaling, B cell activating factor receptor (BAFFR), CD40, and the TLRs (57, 60). When tolerance is effective there is an equal balance between negative and positive selection. In autoimmune diseases, this immune balance is lost and favors negative selection. Excessive amount of BAFF can result in negative selection and consequent produce autoimmunity is depicted in Figure 3. Transitional B cells mature and populate the follicular mature (FM) compartment or the marginal zone (MZ) and germinal centers of LNs and spleen (61). While majority of the autoreactive BCR specificities are deleted by negative selection, some in the MZ compartment survive and may contribute to autoimmunity (56). High levels of BAFF can rescue low affinity autoreactive B cells from negative selections. It is usually believed that more than 90% of autoreactive B cells are eliminated by checkpoints. Alterations in checkpoints have been associated with high levels of autoreactive B cells, T cells, and autoimmune disease (53, 56). During remissions, checkpoints resume normal functions, reduce autoreactive cells, and return to pre-disease or physiological status (62).

### B Cells and Plasma Cells

B cells and plasma cells residing in specific niches, differentiate by a sequential processes, aided by growth and survival factors and their signals. Upon leaving the BM, B cells circulate through PB, reach the LNs and spleen, encounter cognitive antigens, costimulatory signals, and dendritic cells, proliferate and differentiate into plasmablasts and plasma cells (63). Short-lived plasma cells have a lifespan of approximately 3 weeks, while long-lived plasma cells may survive for 6 months or more (63). While majority reside in the BM, a smaller population may reside in the spleen (63). Their longevity is influenced by the microenvironment, including specific survival signals from BM and inflamed tissues (63).

### Autoreactive B Cells

Autoreactive B cells play a significant role in propelling from autoimmunity to autoimmune disease. They act as antigen-presenting cells, interact with T cells in the germinal centers or target tissues, and may undergo somatic hypermutation and class-switch recombination (64). They amplify the inflammatory response and play a significant role in generating autoimmune pathogenic memory B cells (64). Certain subsets of B cells that express specific factors, can convert them to pathogenic autoantibody secreting cells, in the presence of tolerance checkpoints (63, 64). Some of those reported include overexpression of CD19, reduced expression of CD21 (63, 64), upregulation of CD95 and BAFF.

The maintenance of autoimmunity is facilitated, in part by the large plasma cells, prone to apoptosis, but thrive in disease microenvironments, because of survival and growth factors (56). For example, BAFF provides survival signals for immature B cells, mature NFB cells, plasma cells in the BM, and CD27^+^ memory cells (65). In the BM there is a feedback loop between pre B cells and plasma cells. Cytokines such as IL5, IL6, TNFα, and SDF-1a that can modulate inflammation and affect longevity of plasma cells (66). In this survival niche, these long-lived memory plasma cells are frequently protected from the effects of ISA and may play a role in disease progression or relapse (16). During relapse, emergence of CD20^+^CD27^+^ memory cells, in association with costimulatory T cells, provide signals to plasma cells so that they can produce more autoantibodies and cause disease (67). In addition, newly generated naive B cells (CD10^+^/IGD^+^) exiting the BM, with T dependent or independent signals, may have the capacity to undergo differentiation and become autoreactive plasma cells (68–70).

### T Cells

**Figure 4:**
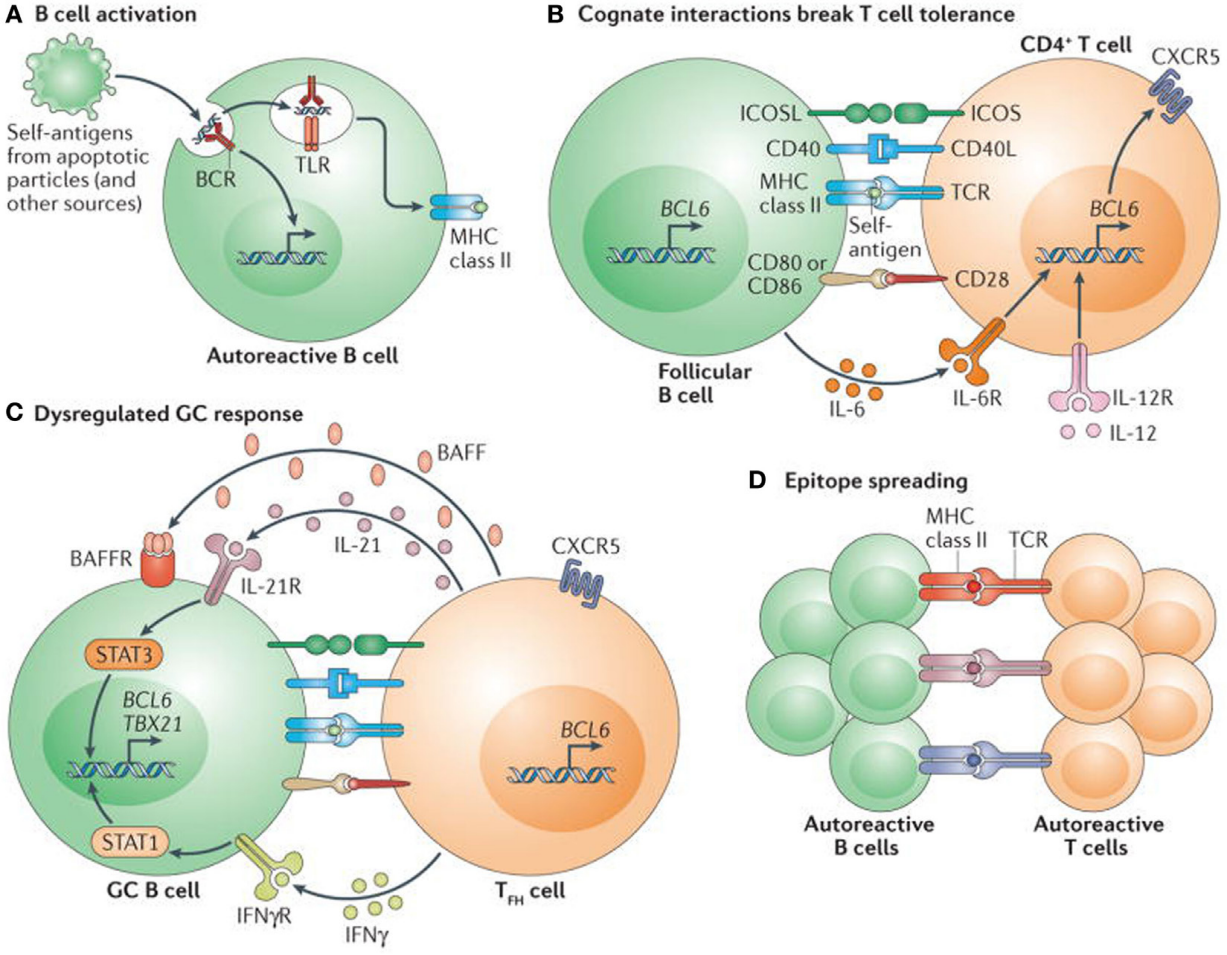
Self reactive B cells initiate autoimmune germinal center (GC) formation by facilitating breaks in T cell tolerance. **(A)** After binding to self-antigen (either soluble or bound to antigen-presenting cells) derived from apoptotic particles or other disease-specific targets, autoreactive B cell receptors (BCRs) traffic nuclear antigens to the endosomal receptors toll-like receptor 7 (TLR7) and TLR9, resulting in initial B cell activation in response to integrated BCR-dependent and TLR-dependent signaling. In parallel, endolysosomal enzymes also process internalized self-antigens (including a broad range of nucleic acid-associated proteins) into peptides for loading onto MHC class II. **(B)** B cells function as antigen-presenting cells to present MHC class II-bound peptides to cognate self-reactive CD4^+^ T cells at the T cell–B cell border of lymphoid follicles. Together with co-stimulatory signals provided by CD80 and/or CD86 and inducible T cell co-stimulator ligand (ICOSL), self-reactive B cells initiate breaks in CD4^+^ T cell tolerance. Activated CD4^+^ T cells subsequently express CXC-chemokine receptor 5 and B cell lymphoma 6 (BCL-6), resulting in their migration to the B cell follicle as early T follicular helper (T_FH_) cells (not shown). Activated B cells also produce interleukin-6 (IL-6), which may facilitate T_FH_ cell differentiation by inducing BCL-6 expression, although this has not yet been directly tested. **(C)** T_FH_ cells promote GC formation through the production of IL-21, which sustains B cell BCL-6 expression and promotes B cell activation, class-switch recombination and plasma cell differentiation. In autoimmune settings, interferon-γ (IFNγ; probably derived from activated CD4^+^ T cells) drives GC formation in a B cell-intrinsic, signal transducer and activator of transcription 1-dependent manner, in part by enhancing BCL-6 expression. IFNγ also promotes B cell-intrinsic expression of the transcription factor T-bet (encoded by *TBX21*), which is required for class-switch recombination to pathogenic IgG2a and IgG2c isotypes, but is redundant for IFNγ-driven GC formation. Although not yet directly tested in autoimmune models, this dysregulated GC response is probably affected by additional cytokines, including B cell-activating factor (BAFF), which promotes the selection of high-affinity GC B cell clones, and IL-12, which facilitates T cell IFNγ production and T_FH_ cell differentiation. **(D)** Iterative interactions between GCB cells and cognate T_FH_ cells within ongoing autoimmune GCs probably result in epitope spreading and the recruitment of additional autoreactive T cell and B cell clones. Abbreviations: BAFFR, BAFF receptor; CD40L, CD40 ligand; ICOS, inducible T cell co-stimulator; IFNγR, IFNγ receptor; IL-6R, IL-6 receptor; TCR, T cell receptor. Reprinted from Ref. (60) with permission from Springer Nature.

T cell tolerance plays a critical and vital role in the generation and persistence of autoimmunity. (Figure 4) The authors have not provided as much information on T cell tolerance, as on B cell tolerance, because of their focus on B cell development and BDT. In the thymus, T cells encounter several self-peptides, in the context of MHC class II gene products, Foxp3 expression on regulatory T cells (CD4^−^, CD25-CTLA4) and proliferate and differentiate into cytotoxic T cells, but most importantly eliminate potentially autoreactive T cells (71). Studies in mice and humans, have demonstrated that T cells proliferate upon contact with dendritic cells and self-antigens, but are promptly eliminated by the processes of deletion or anergy and thus maintain tolerance (71). However, when these T cells are in contact with the inappropriate dendritic cells or an inappropriate self-antigen, they undergo a process of defective deletion or loss of anergy, and consequently became autoreactive and can produce autoimmunity (71).

Autoantibodies can be generated from autoreactive B cells, when activated by T independent signals some of which may act through toll-like receptors. Toll-like receptor 7 (TLR7) has promotional, while TLR9 has a regulatory role (72). Some other molecules that influence this process are CD38, CD40, CD40L, TLR7, 9, IFN-γ, IL6, IL10, IL21, CD86 (72). Dendritic cells often vital in autoimmunity can act independently or in collaboration with several cytokines, some of which are CXL2, 3 BAFF, BAFF-R, FCγR, APRIL, and interferon γ (71, 73).

Interestingly, active T cells can inhibit further T cell stimulation using dendritic cells and IL10 as a mediator, while simultaneously they are known to facilitate, T cell activation through IL6 (74) Therefore, it appears that they can simultaneously exert opposite effects on immune responses, indicating that their biological activities depend on their microenvironment. This has clinical relevance because the clinical course of many autoimmune diseases has multiple stages and phases, and microenvironments may significantly influence them (74).

Recent studies suggest that regulatory B cells (B_regs_) that produce IL10 or TGFβ, capable of preventing or suppressing autoimmunity, may do so by introducing anergy in T cells (74).

The anatomical microenvironment of the germinal centers, considered essential for B cell differentiation and maintenance of homeostasis, may also facilitate induction of effector T cell function (75). B cells can influence maintenance and development of effector CD4^+^ memory T cells, induction of peripheral tolerance, and regulation of the balance of helper T cells (75, 76). In germinal centers, B cells can present antigens to memory T cells and elicit cytokine production (77). They provide a second activation signal to follicular CD4^+^ T cells, previously activated by dendritic cells (75, 76). Consequently, IL4 produced within the germinal centers can provide a microenvironment for Th2 development (77).

## B Cell Depletion Therapy

The concepts that guided initial investigations to use BDT in treating autoimmune disease, were primarily to provide the immune system reinstallation of regulating of emerging autoreactive B lymphocytes and establish a normal B cell repertoire (i.e., restoration of tolerance) (78).

B cell depletion therapy agents consist are hybridomas, produce IgG1 monoclonal antibodies that specifically target the CD20 molecule (78). The presence on mature B cells, but not on stem cells, plasmablasts, and plasma cells (79) significantly influences the pharmacokinetics and biopharmacology of BDT. Its efficacy depends on dosage, diffusion into the tissues, kinetics of elimination, and frequency of administration (80).

### Mechanism of Action

The binding of BDT to the CD20 molecule leads to cell lysis and subsequently their disappearance from PB. Some depletion mechanisms include antibody dependent cellular cytotoxicity (ADCC), complement dependent cytotoxicity, phagocytosis by the reticuloendothelial system, and apoptosis of B cells by crosslinking CD20 molecules (80).

The clinical benefits of BDT were based on the “Road Block Hypothesis” (81). In the road from physiologic to autoimmune state, the autoreactive B cells may play a significant role in producing inflammation and pathogenesis by shifting the repertoire toward antigen-specific autoreactive B cells and BCR. Therefore, the hypotheses proposed that, depleting these autoreactive B cells, could eliminate interactions between costimulatory signals and pro-inflammatory mediators (81). Consequently, the “road” to autoimmunity would be blocked; local inflammation would be eliminated, resulting in clinical recovery.

### Effects on Different Compartments of Immune System

B cells depletion is not universal, differs significant in different compartments of the immune system which has implication on clinical outcomes. 100% depletion is observed in the PB, compared to 32% in the BM, perhaps lower levels in germinal centers and marginal zones of LNs and spleen (82, 83). Furthermore, PB CD20^+^ cells account for approximately 2% of the total B cell population (84). Usually B cells differentiation and maturation occurs in the BM, where stromal cells produce factors promoting their survival and growth (85).

### Role of Memory B Cells

The role of memory B cells in the clinical responses, final clinical outcome, notably relapse, cannot be overemphasized. Clinical response is observed with depletion of CD19^+^, CD27^+^ memory cells from the PB and BM (86). Studies demonstrate that pre-therapy levels of CD27^+^ memory cells may predict clinical response. Better responses are observed in patients with lower levels than with higher levels (87, 88). Clinical response is also influenced by the pre and post treatment levels of long-lived plasma cells and levels of survival factors (87, 88). After BDT infusion, there is a repopulation of naïve B cells that are CD38 high, CD27 high, and sIgD^−^. There is a decrease in the number of non-class-switched (IgD^+^, CD27^+^) and class-switched (IgD^−^, CD27^+^) memory B cells (86, 89). A gradual decrease in levels of naïve B cells and a progressive increase in CD27^+^ memory B cells occur as the pharmacological effects BDT begin to diminish (86).

Patients that do not demonstrate a favorable clinical response to the initial treatment with RTX, may have high levels of plasmablasts/plasma cells. Additional cycles, before complete repopulation occurs, could increase chances of good clinical response (86).

Several patients in clinical remission may have demonstrable levels of autoantibodies, because long-lived plasma cells, unaffected by BDT, produce them (90).

After depletion, return to normal levels is observed in all B cell compartments. Simultaneously, return to balance and appropriate ratios of Th1/Th2, increased numbers of helper T cells and T regulatory (T_reg_) cells occurs (91). Increase in T_regs_ is reported in ITP and systemic lupus erythematosus but not in RA patients, may be because they are simultaneously treated with methotrexate (91–93). This observation puts into focus the effects and influence of CS and ISA used as concomitant therapy with RTX. In many studies, “concomitant therapy” after RTX is continued, albeit in lower dosages.

### Duration of Remission

The duration of clinical remission following BDT depends on known and unknown factors. A key factor is the duration required for CD27^+^ memory B cells to exit the BM, reach the spleen and LNs, and become autoreactive (88). Naïve memory cells are more susceptible than long-lived memory B cells (88). It is reported that, if at 2 years post-RTX, the levels of memory cells are less than 50% of their pretreatment levels, remissions are longer (94, 95). The process of conversions into memory cell phenotype occurs by somatic hyper-mutation, during highly variable periods, and can take up to 6 years after a single cycle of BDT (89). Longer remission occurs when fewer memory cells enter germinal centers of LNs and spleen and become plasmablasts and plasma cells (94).

### Repopulation

Almost complete depletion of CD20^+^ B cells in the PB occurs within 3–7 days after the first infusion of BDT. Repopulation to normal levels in the PB, minimally requires between 6 and 12 months and could be 3 years (84). Repopulation influences clinical course, outcome, need for future therapy, and depends on extent of depletion, clearance of BDT and BM capacity to regenerate (84). A factor that has not received attention is presence of comorbidities, specially coexisting autoimmune diseases.

Formed inside and outside germinal centers, several subpopulations of memory cells, with different phenotypes develop during repopulation (88). In autoimmune diseases, there are greater expansions in IgD^−^IgM^+^CD27^+^ and IgG^−^CD27^+^ phenotypes (96–98), that predominantly use IgG1 and IgG3, potent activators of complement and involved in target killing by ADCC (99).

During the process of repopulation, CD27^+^IgD^−^IgM^−^CD38^+^ plasmablasts undergo differentiation, maturation, somatic mutation, and eventually become plasma cells (100, 101). CD20^−^ plasma cells not detected in the PB during depletion are detected during repopulation (102, 103). Not infrequently, measurable levels of pathological autoantibodies are detected when repopulation reaches pretreatment levels, since long-lived plasma cells, repopulate (104) consequent to increased production of BAFF by the spleen (105). This is a paradoxical effect of BDT. It depletes CD20^+^ B cells, but facilitates the differentiation and growth of short-lived plasma cells to become long-lived plasma cells in the spleen (105–107).

### Relapse After BDT

Rheumatoid arthritis (RA) was the first autoimmune disease, in which multiple trials showed significant clinical benefit after two infusions of 1 g of RTX, 15 days apart, mostly in 6–12 months follow up studies (108). In RA, the high incidence of relapse requires multiple cycles to maintain clinical remissions (12, 15), having influence on cellular and humoral immune responses (74). Incidence of relapse is directly related to the duration of follow-up reported in the study (11). Longer follow-ups have higher incidence, reaching 70–85% (11).

Usually relapse does not occur until repopulation of CD20^+^ B cells reach pretreatment level (86, 101, 109), and with increase in B cells exiting the BM into the microenvironment (90, 110), where with appropriate signals they differentiate into autoreactive B cells (90).

Levels of BAFF and autoreactive plasmablast are high in the PB during active disease (111). BAFF-R expression is reduced on naïve and memory B cells during relapse, regardless of serum BAFF levels (110).

Repopulation of autoreactive memory B cells and/or plasmablasts accompany relapse, and may be predictors of relapse (88). Soluble-free light chains and CD23 affect differentiation of plasmablasts present in early phase of relapse (90, 111). During relapse CD95^+^CD27^+^ cells that produce proinflammatory TNFa and IL10 have higher ratios compared to transitional cell in PB (112).

In the microenvironment of the germinal center, maintenance of memory B cells is independent of T cell interaction (75), but necessary for their differentiation into long-lived plasma cells (75, 113).

Proinflammatory autoreactive B cells in the microenvironment are characterized by the Ki67 marker present on pre B and immature B cells leaving the BM, while kappa-deleting recombination excision circles characterize migrating transitional cells (114, 115). Autoreactive B cells demonstrate high proportions of Hep2 autoreactive antibodies and have high prevalence of T1858, PTPN22 risk alleles (55, 64).

B cell depletion therapy has several serious adverse events, notable infection, due to immune suppression, resulting in septicemia and death (116). IVIg can reduce this risk. Late onset neutropenia associated with pneumonia (12, 116, 117) and cardiac issues (117) is of significant concern.

From a panoramic perspective, some investigators have suggested the following, “the evidence therefore indicates that currently used anti-CD20 treatments do not return the patient to an earlier state of immunity akin to the ‘tabula rasa’ (that is a blank slate), in which all the past levels reminiscent of memory and proves (auto) immune responses have been excised” (100).

Processes vital to central and peripheral tolerance, such as anergy, receptor editing, and deletion among others, are not affected or restored by BDT (118), consequently it cannot eliminate autoimmunity for sustained durations. Eventually, autoreactive B cells with the cooperation of autoreactive T cells produce inflammation giving the microenvironment a pathologic profile and autoimmune disease return (118).

## IVIg Therapy

Intravenous immunoglobulin has been used in treating immuno-deficiencies for more than six decades. It has to be used as monotherapy, or as an adjunct with other drugs, in treating autoimmune and inflammatory disease for several years (17). It influences almost every component of the immune and inflammatory systems, producing multiple beneficial results.

### Mechanism of Action

**Figure 5:**
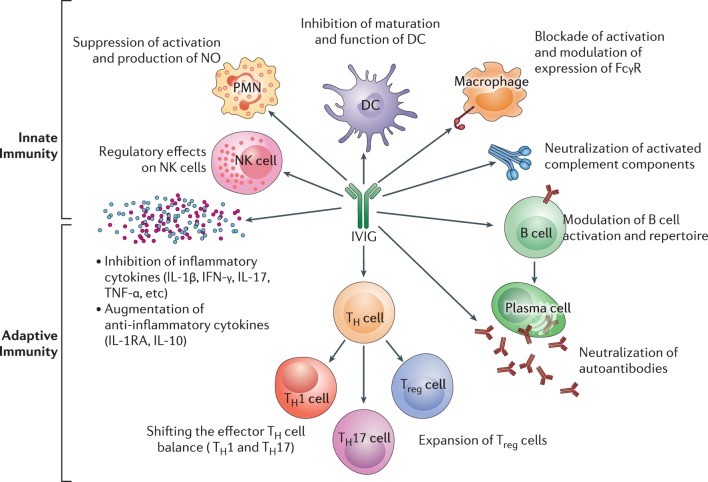
The impact of intravenous immunoglobulin (IVIg) on the innate and the adaptive immune compartments in the context of autoimmune and inflammatory diseases. IVIg inhibits activation and function of various innate immune cells, such as DCs, monocytes, macrophages (M⌽), neutrophils [polymorphonuclear cells (PMN)], and NK cells. It neutralizes activated complement components. In addition, IVIg modulates B-cell functions and plasma cells (Pl), reciprocally regulates T_reg_ cells and effector T cells, such as T_h_1 and T_h_17 subsets and downregulates the production of inflammatory cytokines. Reprinted from Ref. (16) with permission from Oxford University Press.

Intravenous immunoglobulin’s has multiple mechanism of action (17). In Figure 5, the influence of IVIg on innate and adaptive immunity is presented. The (Fab)_2_ fragment and Fc fragment of the molecule, are known to have different functions and effects. The (Fab)_2_ fragment plays a vital role in killing of target cells, the blockade of cell–cell interactions mediated by cell-surface receptors, such as CD95 and CD95 ligand; the neutralization of cytokines; the neutralization of autoantibodies by anti-idiotypic antibodies; and the scavenging of the anaphylotoxins C3a and C5a (17, 18). The Fc portion may play a vital role in Fc-dependent pathways include; the saturation of the FcRn; the expansion of regulatory T (T_reg_) cell populations; the blockade of immune complex binding to low-affinity Fcy receptors (FcyR); the modulation of dendritic cell activation *via* FcyRIII; and the modulation of activating and inhibitory FcyR expression on innate immune effector cells and B cells (19, 20). IVIg regulates FcyRIIIa and IFN-yR2 on circulating dendritic cells and by stimulating production of IL-33 by human macrophages (119). This provides an important anti-inflammatory effect of IVIg through the IL1 receptor antagonist levels (120).

Its anti-inflammatory effects and its ability to regulate immune balance are its most important functions (21–23, 121–125). Therefore, IVIg has the capacity to eliminate clinical autoimmunity, restore a state of tolerance, and reinstate physiologic homeostasis (17, 18).

### Role in Autoimmunity and Autoimmune Disease

Intravenous immunoglobulin can influence repertoire of mature B cells, including their heterogeneity, and consequently plasma cells (17), which affect the persistence of autoimmunity, and possibly its elimination (24, 126–129).

Intravenous immunoglobulin facilitates the migration of immature plasmablasts and naïve plasma cells from BM into PB and eventually the microenvironment of diseased tissue (129, 130). These cells differentiate into CD138^+^ plasma cells that produce normal or physiologic antibodies that play a critical role in restoring immune balance (18). In the diseased microenvironment, previously present pathogenic autoantibody producing plasma cells compete with these new normal plasma cells for survival and growth factors (131). Positive clinical response to IVIg correlates with increased number of normal CD138^+^ plasma cells (130).

The effects of IVIg on regulatory T cells (T_regs_) eventually decrease inflammation resulting from Th1 and Th17 cells (23). IVIg imposes a tolerogenic state to the dendritic cells accompanied by an expansion of antigen-specific T_regs_ which are ultimately capable of decreasing inflammation in the microenvironment (23, 132).

Several lines of evidence indicate that IVIg establishes a physiologic homeostasis in tissue environments, by multiple mechanisms such as anti-idiotypic antibodies (127, 133, 134), increasing the population of T_regs_ and normal BCR, the TGF_B_ receptor of T and B cells, downregulating TNFα. IL-1, 2, 3, 4, 5, 6, 8, 11, and 17 and CXL2, 3, and 5 decreasing amounts of BAFF/APRIL and modulating FcyR (18–20, 119, 127, 133–138). Decrease in tissue inflammation occurs because IVIg maneuvers the functionally aberrant lymphocytes, neutrophils, macrophages, and other cells toward their normal physiologic states (18–20, 119, 127, 134–138). IVIg facilitates the development and proliferation of regulatory T_regs_ and B_regs_ (20, 23, 127, 132, 134–139).

### Influence on Checkpoints

The role of checkpoints is crucial in maintaining the state of tolerance and is detailed in Figure 2 (56). Through the process of somatic mutation in naïve B cells, IVIg enhances the expression of IgD on the cells surface (140), which is implicated in the induction of anergy. Continued anergy eventually silences autoreactive B cells (56, 64). Receptor editing of BCR in autoreactive B cells is facilitated by the interaction between IVIg and CD22 (141). IVIg influences normal B cell by enhancing Vh germ line usage, and VDJ recombination process, which indirectly affects autoreactive B cells (142). IVIg promotes increased usage of Vh3–30 and 3–23 gene segments during the VDJ recombination process. This promotes the increased production of a normal B cell repertoire (142–144). It induces apoptosis of many cells *via* either Fas–FasL pathways or through anti-idiotypic antibodies (145, 146). Thus the cumulative evidence demonstrates that IVIg can induce and promote processes that mimic the actions and functions of physiologic checkpoints and thus has the capacity and efficacy to eliminate autoreactive T and B cells (57, 59).

### Role of IVIg in Immunoprophylaxis

One of the most important aspects and secondary benefits of the Ahmed protocol is the role of IVIg in providing immunoprophylaxis, as evident by the fact that none of patients, in their studies had serious infections, were hospitalized or died from infectious etiologies (29, 41–44). Prolonged B cell depletion can induce immunosuppression by several mechanisms (147). In RA, it has been reported that 10% patients have hypogammaglobulinemia after first course of RTX and 30% after fourth course (148), becoming a risk factor for infection (147). RTX induced prolonged B cell depletion impairs T cell-mediated immunity with decreased risk for viral and fungal interferons (147). The issue of infection during immunosuppression is more significant in blistering disease, because denuded epithelium is more susceptible to infection. Indeed currently, the commonest cause of death in blistering diseases is infection (149).

There is significant body of evidence that documents the ability of IVIg therapy to prevent and fight serious infections and potentially fatal infections (150). An extensive or detailed validation of this is beyond the scope of this concept paper. Examples of such situations are as follows. Patients with RTX-induced hypogammaglobulinemia have serious, multiple, or recurrent infection for which IVIg therapy has been used (151–153). IVIg is a valuable therapeutic agent in several serious infections (154–156) and its use resulted in decrease of duration of hospitalization (157). It is considered standard therapy in patients with sepsis (150, 158, 159) including neonatal sepsis (160) and especially in post-surgical sepsis (161–163). It has been frequently used to treat immunosuppression in patients with malignancy, wherein the cancer itself or to its treatment produced it (164), and with significant benefits in multiple myeloma (165).

Intravenous immunoglobulin is also routinely used in protocols for bone marrow or solid organ transplants (166, 167). A meta-analysis of nine randomized trials, involving 435 patients using several objective parameters for assessment, in patients with severe sepsis and septic shock, IVIg not only was more effective clinically but also more cost-effective (168).

In Table 2, the authors have presented a synopsis of how the various compartments of the immune system are affected in autoimmunity. It is not all inclusive or exhaustive. Its purpose is to demonstrate some of the influence of BDT and IVIg on these compartments.

**Table 2:** Mechanisms of immuno-regulation in the microenvironment in an autoimmune disease state and post-therapy.

	Microenvironment, tolerance, checkpoints	Ig, autoAbs, BCR, and Fc receptor	B cells, memory cells, and plasma cells	Cellular and complement pathways	Cytokines
Autoimmunity (lifelong)	– Chronic inflammation – Loss tolerance – Defective checkpoints	– Increased IgG class switching – Elevated levels autoAbs – Formation immune complexes – Activation FcγRI/FcγRIII loss regulatory FcγRIIB	– Increase autoreactive B cells – Induction of short- and long-lived autoreactive memory and plasma cells	– Increased neutrophil/monocytes/macrophages activation – Activation complement cascade – Activation autoreactive T cells – Imbalance ratio of Th/Tregs Cells	Inflammatory: – IL1–10, INFγ, TNFa, TGF-b CXCL3, CXCL5, CCL13, XCL2 IL10: – Loss of protective effect – Production B cell autoAbs – Favors class switch to IgG4
B cell depletion (repeated infusions)	– Rapid, temporary depletion CD20^+^ B cells decrease inflammation – No effect on tolerance – No effect on checkpoints	– Downregulation BCR – Decreased autoAbs levels – Inhibition of FcRs – Repeated infusion reduces Serum IgM, IgA, and IgG	– Increase transitional cells – Increase Bregs – Decrease short-lived autoreactive cells – Expansion autoreactive memory B-cells – No effect on long-lived autoreactive plasma cells	– Neutropenia – Decreased monocytes/macrophage/DC activity – Complement consumption – Restoration Th1/Th2 ratio – Increase Tregs – Inhibit CD4 + T cells	– Decreased IL activity – Increased BAFF/APRIL levels – Increased transitional Anti-inflammatory IL-10
Intravenous Ig (repeated infusions)	– Sustained, decreased inflammation – Restores tolerance – Mimics checkpoints	– Inhibit FcγRI/FcγRIII – Upregulate FcγRIIB – Edit autoreactive BCR – Deletes autoreactive BCR – Deactivate autoreactive BCR – Increased production new non-self-reactive IgG, IgM	– Inhibition autoreactive B-cell differentiation – Induction B-cell apoptosis – Regulation B-cell subsets – Production of new, naïve non-autoreactive plasma/memory cells	– Neutrophil apoptosis – Reduction complement levels – Modulation CD4 + – T cell differentiation – Suppress Ag-specific T cells – Upregulation Tregs population	– Inhibition selective IL activity, – Decrease BAFF/APRIL levels – Increase regulatory anti-inflammatory IL-10 production

*Abs, antibodies; Ag, antigen; APRIL, a proliferation ligand; BAFF, B cell activating factor; BCR, B cell receptor; Bregs, B regulatory cells; DC, dendritic cell; Ig, immunoglobulin; IL, interleukin; R, receptor; Th, T helper cell; Treg, T regulatory cell*.

## Selected Recent Studies and Relationship to “Concept”

Several recently published studies have impact and influence on the “concept.” However, two publications warrant mention because of their content and immediacy of impact.

First is a recent study involving 90 untreated PV patients, by 36 investigators, from 25 centers in France, in which RTX is recommended as first-line treatment (169). The control group had 44 patients, who received only prednisone, dose of 1 mg/kg/day, for moderate disease, for 12 months, and 1.5 mg/kg/day, for severe disease, for 18 months. The study group of 46 patients was treated as follows. Initially, moderately severe patients received 0.5 mg/kg/day for 3 months and patients with severe diseases got 1.0 mg/kg/day for 6 months. They received 1 g of RTX at day 1 and 15, followed two prophylactic doses of 500 mg each, at month 12 and 18. This was done, although in an earlier report, the lack of benefit from “prophylactic” use of RTX in preventing relapses had been demonstrated (170). A statistically significant difference in benefits was observed in the RTX (study) group.

There are certain grave concerns regarding this study. The initial design of 2 years follow-up was extended to 3 years, with only one visit at month 36. Surprisingly, 79 of 90 patients (88%) with severe disease were never treated. Usually patients in countries with or without socialized medicine, with PV, would have been treated soon, and not have allowed to progress to untreated severe disease.

Incidence of relapse is vital to any RTX study on PV and reflects its validity and utility. Information on relapses is presented at multiple sites. The authors state that at month 24, 11 out of 44 patients (25%) had relapses, 4 (10%) of which were severe and 7 (15%) were moderate. Careful reading showed that 8 of 11 occurred in 6–12 months and three during 12–24 months. However, at month 36 only 2 of 41 patients (2%) had a relapse. Yet the authors report that 41 of 44 (89.8%) patients were in complete remission, off therapy at month 24. What is lacking is information on duration and treatment of the relapses, since there is no mention of additional RTX treatment. In the patients who relapsed within 6–12 months, were their B cells completely deleted by the two RTX infusion, prior to their developing a relapse? Did their B-cells repopulate within 6–12 months? How successful is a therapy when 25% of patients, relapses in less than 1 year? Since relapses were accompanied with increase in anti-desmoglein autoantibody titers, the critical question is, after initial RTX therapy did anti-desmoglein antibodies decrease, disappear, or remain unchanged. This would also be a valuable index of efficacy. The relapses in this study further support the inability of the “prophylactic” use of RTX to prevent relapses.

The issue of relapse and its management is critical to RTX therapy and to physicians using it. In these PV patients more detailed and clear information was needed.

It is difficult to duplicate or verify such a study based on patient selection. Finding 79 patients with severe untreated disease is a daunting task. Similarly, administering only high dose systemic corticosteroids, without any ISA is unlikely, especially when their significant and catastrophic adverse events are known and especially in patients with severe disease. In most instances, patients, their relatives, or their primary care physicians would not permit this option. It is also unclear that how many patients had more than one serious adverse event. The authors report that from a total of 80 patients, 20 patients (25%) had significant infections, 10 patients (13%) cardiovascular disorders, 6 patients (8%) psychiatric disorders, 10 patients (13%) bone disorders including fractures and osteoporosis 17 patients (21%) diabetes and endocrine disorders, while 13 patients (16%) developed steroid myopathy. It appears that these data were collected at month 24, and are very significant and concerning. What about month 36?

The manufacturer provided the drug. Based on this specific study, RTX has been granted Expedited Review or Fast Track Approval by the Food and Drug Administration of the US. Most investigators would consider a confirmation of this study in the US, essential. In studies with long-term follow-up, multiple relapses are reported (11). It would be important to know of the 44 study group, French patients develop relapses on longer observation periods. In spite of these comments, it needs to be recognized that this is the only randomized controlled trial (RCT) examining the efficacy and safety profile of RTX in PV. Recent studies indicate multiple infusions of BDT are necessary for rheumatoid arthritis patients to maintain a comfortable clinical condition (92). Consequences of large numbers of infusion, on the immune and inflammatory systems, could possibly emerge 20–30 years later.

An interesting and relevant clinical trial is currently in progress. A total of 124 patients with moderate to severe PV will be enrolled at 60 centers worldwide, to evaluate the efficacy and safety of RTX versus mycophenolate mofetil. It will involve a 52-week double blind treatment period and a 48-week post-treatment discontinuation safety follow-up period. Since the follow-up period is limited, it may not address its impact on the clinical course, especially production of long-term sustained clinical remissions or the issue of later relapses. Information on this clinical trial is available at clinicaltrials.gov identifier NCT02383589.

The second study, from the October 2017 issue of Frontiers in Immunology (84) examines what has been learnt from therapies that target CD20 and future trends. Most importantly, it addresses a vital issue, the need to enhance the efficacy of current BDT. This efficacy depends on its ability to penetrate lymphoid tissues (171). Studies in mice and primate indicate that B cell depletion in the BM, spleen, and lymphoid tissues frequently require larger amount of anti CD20 antibody. If not eradicated, there is a potential for such sites to act as reservoirs, from where autoreactive B cells can emerge leading to relapse (84). This may account for inability of a single cycle of RTX, to produce long-term remissions and also cause for frequent relapses in many patients (84). Cumulatively, these studies explain why frequent multiple doses given initially are more effective. Indirectly they also provide support for the initial and 1-year systematic use of RTX in the Ahmed Protocol.

Information on enhancing efficacy of BDT therapies is still evolving. Glycoengineering and Fc engineering have shown benefits (84). Several new immunomodulatory molecules are being developed for use with anti CD20 therapy. Some of these are STING antagonist which increases expression of activated FcγR’s crucial for antibody mediated therapy (84). Ibrutinib, an irreversible inhibitor of Btk, idelalisib targets the delta form of lipid kinase phosphoinositide-3-kinase expressed on leukocytes, Ventoclax an inhibitor that targets Bcl-2 (84). Bispecific antibodies are emerging (84). Investigators have combined antibodies to CD19 and CD3, CD20 and CD22, and a tribody which combine two CD20 antibodies with antibody to Fcγ RIIIA (84). Lenalidomide combined with anti-CD20 has greater benefit than anti-CD20 alone (84).

Decrelizumab as an anti-CD20 antibody has been effective in progressive multiple sclerosis (MS) and Ublituximab, a glycoengineering anti-CD20 mAb also helps MS patients. Some investigators recommend antibodies to BAFF to be more effective than RTX alone in autoimmune disease (84).

Autoreactive B cells and T cells have multiple participants during their development and functional processes. Blocking a single or limited number, may cause the human body to find the alternative pathways to bypass the block. Since IVIg influences many pathways of the immune and inflammatory system. (Table 1), it is entirely possible that IVIg may already contain some of these pharmacologically active agents under investigation and more. Their concentration may be low and variable. Further such studies on IVIg are, therefore, warranted.

## Synergistic Effect of IVIg with RTX

Based on the Ahmed protocol presented in this manuscript (Figure 1), the preceding discussion provides some mechanisms that account for the positive clinical outcome and prolonged remissions.

Rituximab eliminates autoreactive B cells from the PB shortly after its initiation. As BM and possibly the spleen and LNs expunge their autoreactive cells, subsequent periodic infusions eliminate them. Reduction of inflammation in the microenvironment, results from elimination of autoreactive B cells and proinflammatory mediators. These processes were enhanced by the anti-inflammatory effects of IVIg used simultaneously. This process continues during the B cell depletion phase. Inspite of lack of B cells, and prolonged immune suppression, there were no serious infections, because of immunoprophylaxis was provided by IVIg. In addition, when the effects of RTX begin to wear off, IVIg exerts immune restoration by increasing T_reg_, B_reg_, macrophages, dendritic cells, and promoting more CD138^+^, normal plasma cells. It decreases population of autoreactive B cells in the microevironment, decreases BAFF, other growth factors, neutralizes cytokines like IL4, IL6, and IL10 among others. Anti-HLA antibodies could reduce presentation of autoantigens to T cell receptors. In the reduction and absence of inflammation, the tissue microenvironment now has the opportunity to return to physiologic homeostasis, since IVIg continued in phase 3 of the protocol, accomplishes this by mimicking the function of checkpoints. In doing so, IVIg reduces emergence of autoreactive B and T cells, possibly decreasing chance for relapse. RTX’s inability to influence checkpoints is compensated by IVIg. The gradual withdrawal of IVIg allows the dysfunctional immune regulation to slowly return to normality until fully restored, and retain it for the foreseeable future.

## Future Study Design

One of the objectives of this concept paper is to stimulate investigators to treat larger cohorts of patients with autoimmune blistering diseases with this combination treatment (Ahmed protocol), and investigate its potential benefit in other autoimmune diseases. Patients who are non-responsive to conventional therapy should be studied first, especially those with recalcitrant diseases. Defined inclusion criteria are essential. Four groups with reasonably similar disease severity would be required (i) RTX^+^ IVIg, (ii) RTX only, (iii) IVIg only, and (iv) CS and ISA. Careful monitoring of clinical disease with an objective scoring system, repeated serological testing, evaluation of lymphocytes subsets in PB at pre-determined time interval, memory B cells (CD27), T_regs_, and B_regs_ plasma cells and regular assessment of several serological markers, such as BAFF, BAFFR, IL1, ILRA, IL4, IL6, IL10, and others. Likewise serum IgG, IgA, and IgM should be evaluated as designated intervals. When possible, various microarrays, proteomics, and other developing assays and technologies could be used to distinguish responders from non-responders. It would be essential to have the number of patients in each category sufficient for statistical analysis. One of the most important and key element would be a follow-up of 5 years or more. Without a long follow-up, the impact of the treatment on the clinical course and relapse cannot be completely assessed. The authors realize that obtaining funding for such a study could be a great challenge.

## Conclusion

In conclusion, the authors consider that the combination of IVIg and BDT (Ahmed Protocol) could be a very valuable modality to treat patients with autoimmune diseases, especially those who are non-responsive to conventional immunosuppressive therapy or to BDT, and especially those with recalcitrant disease. The authors are not recommending this as standard of care or first-line therapy. Instead, it should be a treatment of last resort. Its initial and limited use has demonstrated that in patients with recalcitrant severe wide spread autoimmune mucocutaneous diseases, prolonged sustained disease and drug-free remissions, without relapse, infections, mortality, or hospitalization have been reported. The authors cannot predict whether factor(s) that initiated or precipitated autoimmune disease cannot recur in the future, or that reversal of autoimmunity would last lifelong. These patients enjoy a high quality of life they had never experienced, nor such a complete remission. These clinical observations provide the foundation and opportunity to conduct research which could produce meaningful insights into central and peripheral tolerance and mechanism for its restoration. Clinical recovery, accompanied by serological and immuno-pathologic remission, persistent normal levels of B cells, and lack of other immune abnormalities, would strongly suggest that, in this patient population, combined treatment with RTX and IVIg, possibly resulted in the reversal of autoimmunity and autoimmune disease.

## Author Note

Since the submission and acceptance of this manuscript in May 2018, the Food & Drug Administration of the US has approved the use of rituximab in the treatment of pemphigus vulgaris.

## Author Contributions

ARA and SK: both authors contributed equally to the entire process.

## Conflict of Interest Statement

The authors declare that the research was conducted in the absence of any commercial or financial relationships that could be construed as a potential conflict of interest.
